# Supplementary material for: Fungal Osteomyelitis: A Systematic Review of Reported Cases
Source: Microorganisms. 2023 Jul 17;11(7):1828. doi: 10.3390/microorganisms11071828 (PMC10383745; doi:10.3390/microorganisms11071828)
Supplement: Supplementary file 1 [file microorganisms-11-01828-s001.zip › microorganisms-2454768-supplementary/Supplementary Table S5.pdf]

Supplementary Table S5: patients characteristics divided according to the etiological agent. Numerical variables are presented as median and range, while categorical variables as counts and percentage. CGD: chronic granulomatous disease; CVC: central venous catheter; IDU: intravenous drug users.

	Etiological agent				
	Aspergillus N = 414	*Blastomyces* N = 47	*Candida* N = 294	*Coccidioides* N = 82	*Cryptococcus* N = 93
Age, years	45 [22 - 63]	28 [15 - 43]	53 [37.3 - 66.7]	32 [23.7 - 46]	46 [33 - 62]
**Gender**					
Male	295 (71.2)	29 (61.7)	192 (65.3)	64 (78)	56 (60.2)
Female	119 (28.7)	18 (38.3)	102 (34.7)	18 (22)	37 (39.8)
**Site of infection**					
Basicranium	30 (7.4)	2 (4.2)	9 (3.1)	-	2 (2.1)
Extremities (no foot)	68 (16.4)	28 (59.6)	39 (13.3)	38 (46.3)	33 (35.5)
Foot	9 (2.2)	3 (6.4)	5 (1.7)	8 (9.7)	4 (4.3)
Other cranium bones	42 (10.1)	8 (17)	11 (3.7)	4 (4.9)	12 (12.9)
Pelvis and hip	14 (3.4)	-	20 (6.8)	13 (15.3)	6 (6.4)
Ribs and sternum	53 (12.8)	1 (2.1)	25 (8.5)	14 (17.1)	18 (19.3)
Shoulder	8 (1.9)	-	4 (1.4)	6 (7.3)	7 (7.5)
Splanchnocranium	149 (36)	1 (2.1)	68 (23.1)	5 (6.1)	4 (4.3)
Vertebrae	125 (30.2)	16 (34)	138 (46.9)	29 (35.4)	23 (24.7)
**Risk factors**					
Bedsores					
CGD	3 (0.7)	-	4 (1.4)	-	1 (1.1)
COVID-19	13 (3.1)	-	-	-	-
CVC	40 (9.7)	-	39 (13.3)	-	-
Diabetes mellitus	23 (5.5)	-	64 (21.8)	-	-
Fungal infection	121 (29.2)	1 (2.1)	87 (29.6)	1 (1.2)	19 (20.4)
elsew.	84 (20.3)	16 (34)	63 (21.4)	32 (39)	19 (20.4)
Fungemia	26 (6.3)	-	49 (16.7)	1 (1.2)	4 (4.3)
HIV	6 (1.4)	-	8 (2.7)	-	4 (4.3)
Hospitalised < 6	45 (10.9)	4 (8.5)	56 (19)	2 (2.4)	9 (9.7)
months	16 (3.9)	1 (2.1)	30 (10.2)	-	1 (1.1)
IDU	81 (19.6)	-	66 (22.4)	3 (3.6)	8 (8.6)
Oncohaematological	78 (18.8)	1 (2.1)	62 (21.1)	7 (8.5)	24 (25.8)
Other immunodepr.	5 (1.2)	-	21 (7.1)	-	-
Parental nutrition	7 (1.7)	1 (2.1)	17 (5.8)	1 (1.2)	-
Prosthesis	52 (12.6)	-	24 (8.2)	1 (1.2)	8 (8.6)
Transplanted	104 (25.1)	8 (17)	95 (32.3)	9 (10.9)	14 (15.1)
Surgery / local lesion					
**Diagnosis**					
Empirical	5 (1.2)	2 (4.2)	4 (1.4)	-	-
Isolation	319 (77.1)	43 (91.5)	258 (87.7)	79 (96)	91 (97.8)
Histological	325 (78.5)	40 (85.1)	203 (69)	65 (79.3)	78 (83.9)
Other microbiological m.	30 (7.2)	-	10 (3.4)	42 (51.2)	41 (44.1)
Radiological	280 (67.6)	43 (91.5)	228 (77.6)	72 (87.8)	86 (92.5)
**Time to diagnosis**					
0 – 1 month	80 (19.3)	12 (25.5)	56 (19)	14 (17.1)	32 (34.4)
1 – 6 months	128 (30.9)	16 (34)	148 (50.3)	34 (41.5)	47 (50.5)
> 6 months	32 (7.7)	5 (10.6)	18 (6.1)	7 (8.5)	10 (10.7)
**Outcome**					
Death	65 (15.7)	-	32 (10.9)	3 (3.6)	7 (7.5)
Recovery	304 (63.4)	44 (93.6)	212 (72.1)	50 (61)	81 (87.1)
Recurrence/chronicity	55 (13.3)	3 (6.4)	29 (9.9)	10 (12.2)	6 (6.5)
**Treatment**					
Duration, moths	4 [0 36], 4*	6 [0.25 24]	3 [0 16], 3*	4.5 [0.75 29], 5*	3.5 [0.25 24], 3*
Antifungal 1	327 (79)	47 (100)	192 (65.3)	58 (70.7)	88 (94.6)
Antifungal ≥2	225 (54.3)	36 (76.6)	113 (38.4)	43 (52.4)	48 (51.6)
Empirical	12 (2.9)	2 (4.2)	4 (1.4)	1 (1.2)	1 (1.1)
Surgery	315 (76.1)	29 (61.7)	182 (61.9)	34 (41.5)	59 (63.4)

Supplementary Table S5: continue.

	Etiological agent				
	Fusarium N = 44	*Histoplasma* N = 14	*Mucormicosis* N = 240	*Paracoccidioides* N = 31	*Phaeohyphomycosis* N = 5
Age, years	40 [18.5 - 55]	31.5 [19 - 51.7]	49 [37 - 60]	37 [21 - 47]	36 [34 - 61]
**Gender**					
Male	27 (61.4)	9 (64.3)	172 (71.7)	22 (71)	2 (40)
Female	17 (38.6)	5 (35.7)	68 (28.3)	9 (29)	3 (60)
**Site of infection**					
Basicranium	1 (2.3)	-	22 (9.2)	-	1 (20)
Extremities (no foot)	12 (27.3)	14 (100)	23 (9.6)	16 (51.6)	3 (60)
Foot	8 (18.2)	1 (7.1)	5 (2.1)	1 (3.2)	1 (20)
Other cranium bones	5 (11.4)	1 (7.1)	22 (9.2)	-	-
Pelvis and hip	-	3 (21.4)	1 (0.4)	2 (6.4)	-
Ribs and sternum	-	3 (21.4)	1 (0.4)	2 (6.4)	-
Shoulder	-	-	-	7 (22.6)	1 (20)
Splanchnocranium	15 (34.1)	1 (7.1)	190 (79.2)	-	-
Vertebrae	7 (15.9)	1 (7.1)	10 (4.2)	1 (3.2)	-
**Risk factors**					
Bedsores	3 (6.8)	-	3 (1.2)	-	-
CGD	2 (4.5)	-	1 (0.4)	-	-
COVID-19	-	-	56 (23.3)	-	-
CVC	18 (40.9)	-	18 (7.5)	-	-
Diabetes mellitus	21 (47.7)	1 (7.1)	142 (59.2)	-	3 (60)
Fungal infection elsew.	14 (31.8)	3 (21.4)	14 (5.8)	19 (61.3)	2 (40)
Fungemia	12 (27.3)	1 (7.1)	9 (3.7)	-	-
HIV	-	2 (14.3)	-	-	-
Hospitalised < 6 months	5 (11.4)	4 (28.6)	18 (7.5)	-	2 (40)
IDU	-	-	2 (0.8)	-	-
Oncohaematological	33 (75)	1 (7.1)	36 (15)	-	-
Other immunodepr.	15 (34.1)	2 (14.3)	40 (16.7)	-	2 (40)
Parental nutrition	5 (11.4)	-	4 (1.7)	-	-
Prosthesis	3 (6.8)	3 (21.4)	2 (0.8)	-	-
Transplanted	15 (34.1)	2 (12.3)	24 (10)	-	2 (40)
Surgery / local lesion	11 (25)	5 (35.7)	63 (26.2)	-	2 (40)
**Diagnosis**					
Empirical	1 (2.3)	-	8 (3.3)	-	-
Isolation	39 (88.6)	12 (85.7)	148 (61.7)	10 (32.2)	5 (100)
Istological	35 (79.5)	13 (92.8)	195 (81.2)	13 (41.9)	4 (80)
Other microbiological m.	-	4 (28.6)	-	-	-
Radiological	13 (29.5)	12 (85.7)	122 (50.8)	13 (41.9)	5 (100)
**Time to diagnosis**					
0 – 1 month	6 (13.6)	7 (50)	48 (20)	6 (19.3)	3 (60)
1 – 6 months	7 (15.9)	2 (14.3)	67 (27.9)	3 (9.7)	-
> 6 months	1 (2.3)	4 (28.6)	8 (3.3)	2 (6.4)	2 (40)
**Outcome**					
Death	14 (31.8)	1 (7.1)	25 (10.4)	-	-
Recovery	26 (59.1)	13 (92.8)	183 (76.2)	10 (32.2)	5 (100)
Recurrence/chronicity	1 (2.3)	5 (35.7)	14 (5.8)	-	1 (20)
**Treatment**					
Duration, moths	2 [0.3 48]	8 [1 16], 4*	3 [0 17], 2*	12 [6 24]	6 [6 12], 1*
Antifungal 1	16 (36.4)	13 (92.8)	138 (57.5)	12 (38.7)	4 (80)
Antifungal ≥2	7 (15.9)	9 (64.3)	100 (41.7)	-	3 (60)
Empirical	1 (2.3)	1 (7.1)	10 (4.2)	-	1 (20)
Surgery	29 (65.9)	9 (64.3)	195 (81.2)	1 (3.2)	5 (100)

Supplementary Table S5: continue.

	Etiological agent	
	Scedosporium<br>N = 77	Other<br>N = 110
Age, years	43 [21 - 57]	46 [32.5 - 52]
Gender		
Male	59 (64.9)	81 (73.6)
Female	27 (35.1)	29 (26.4)
Site of infection		
Basicranium	4 (5.2)	3 (2.7)
Extremities (no foot)	20 (26)	35 (31.8)
Foot	9 (11.7)	5 (4.5)
Other cranium bones	9 (11.7)	6 (5.5)
Pelvis and hip	1 (1.3)	6 (5.5)
Ribs and sternum	2 (2.6)	3 (2.7)
Shoulder	2 (2.6)	1 (0.9)
Splanchnocranium	20 (26)	58 (52.7)
Vertebrae	24 (31.2)	11 (10)
Risk factors		
Bedsores	3 (3.9)	3 (2.7)
CGD	1 (1.3)	-
COVID-19	-	39 (35.5)
CVC	17 (22.1)	18 (16.4)
Diabetes mellitus	21 (27.3)	54 (49.1)
Fungal infection elsew.	24 (31.2)	19 (17.3)
Fungemia	13 (16.9)	11 (10)
HIV	1 (1.3)	2 (1.8)
Hospitalised < 6 months	10 (13)	7 (6.4)
IDU	6 (7.8)	-
Oncohaematological	33 (42.9)	32 (29.1)
Other immunodepr.	16 (20,8)	20 (19.2)
Parental nutrition	4 (5.2)	5 (4.5)
Prosthesis	3 (3.9)	4 (3.6)
Transplanted	22 (28.6)	17 (15.5)
Surgery / local lesion	21 (27.3)	14 (12.7)
Diagnosis		
Empirical	2 (2.6)	1 (0.9)
Isolation	71 (92.2)	102 (92.7)
Istological	57 (74)	94 (85.4)
Other microbiological m.	3 (3.9)	8 (7.3)
Radiological	47 (61)	70 (63.6)
Time to diagnosis		
0 – 1 month	15 (19.5)	19 (17.3)
1 – 6 months	29 (37.7)	55)50)
> 6 months	6 (7.8)	2 (1.8)
Outcome		
Death	15 (19.5)	15 (13.6)
Recovery	45 (58.4)	84 (76.4)
Recurrence/chronicity	6 (7.8)	10 (9.1)
Treatment		
Duration, moths	6 [0 24], 3*	4 [0.5 24], 1*
Antifungal 1	33 (42.8)	41 (37.3)
Antifungal ≥2	17 (22.1)	25 (22.7)
Empirical	5 (6.5)	6 (5.5)
Surgery	48 (62.3)	90 (81.8)

(*) indicates how many patients require a chronic treatment.
