# Supplementary material for: Fungal Osteomyelitis: A Systematic Review of Reported Cases
Source: Microorganisms. 2023 Jul 17;11(7):1828. doi: 10.3390/microorganisms11071828 (PMC10383745; doi:10.3390/microorganisms11071828)
Supplement: Supplementary file 1 [file microorganisms-11-01828-s001.zip › microorganisms-2454768-supplementary/Supplementary Table S6.pdf]

Supplementary Table S6. Results of logistic regressions showing association of outcome (death, recovery, chronicity/recurrence) with variables of interest after elimination of articles of insufficient quality (54 in total, 3 for the death outcome, 32 for the recovery outcome, 1 for chronicity/recurrence outcome).

**Outcome: Death (n=50)**			
**Variable**	**OR**	**95%CI**	**pvalue**
Surgical treatment	0.672	[0.365 1.270]	0.21
Treatment duration, months	0.543	[0.413 0.679]	**<0.001**
Time to diagnosis			
0 – 1 month	0.713	[0.381 1.301]	0.28
1 – 6 months	1.783	[0.987 3.247]	0.055
> 6 months	0.692	[0.256 1.579]	0.42
Immunocompromised	1.612	[0.874 3.089]	0.135
Site of infection			
Basicranium	1.398	[0.458 3.514]	0.51
Extremities (except foot)	0.496	[0.228 0.986]	0.058
Foot	-	-	-
Other cranium bones	2.21	[0.983 4.619]	**0.042**
Pelvis and hip	0.197	[0.011 0.942]	0.113
Ribs and sternum	0.806	[0.297 1.847]	0.638
Shoulder	0.482	[0.022 2.443]	0.484
Splanchnocranium	0.840	[0.309 1.929]	0.705
Vertebrae	1.698	[0.898 3.131]	0.094
Etiological agents			
Aspergillus	2.713	[1.484 4.950]	**0.001**
Blastomyces	-	-	-
Candida	0.692	[0.292 1.457]	0.364
Coccidioides	0.426	[0.023 2.135]	0.412
Cryptococcus	0.432	[0.127 1.109]	0.119
Fusarium	4.965	[0.993 20.89]	**0.031**
Histoplasma	0.649	[0.035 3.406]	0.682
Mucormicosis	1.356	[0.492 3.191]	0.516
Paracoccidioides	-	-	-
Phaeohyphomycosis	-	-	-
Scedosporium	0.803	[0.187 2.376]	0.726
Other	1.322	[0.301 4.094]	0.663
**Outcome: Recovery (n=367)**			
**Variable**	**OR**	**95%CI**	**pvalue**
Surgical treatment	1.490	[0.874 2.501]	136
Treatment duration, months	1.141	[1.057 1.248]	**0.002**

Supplementary Table S6. Results of logistic regressions showing association of outcome (death, recovery, chronicity/recurrence) with variables of interest after elimination of articles of insufficient quality (54 in total, 3 for the death outcome, 32 for the recovery outcome, 1 for chronicity/recurrence outcome).

Time to diagnosis			
0 – 1 month	1.058	[0.643 1.756]	0.825
1 – 6 months	0.966	[0.587 1.604]	0.894
> 6 months	0.886	[0.469 1.780]	0.722
Immunocompromised	0.780	[0.464 1.291]	0.34
Site of infection			
Basicranium	0.782	[0.344 2.016]	0.581
Extremities (except foot)	1.644	[0.945 2.981]	0.088
Foot	6.434	[1.343 115.55]	0.069
Other cranium bones	0.616	[0.312 1.288]	0.176
Pelvis and hip	1.402	[0.575 4.207]	0.496
Ribs and sternum	0.860	[0.446 1.773]	0.667
Shoulder	0.965	[0.305 4.262]	0.956
Splanchnocranium	1.065	[0.533 2.322]	0.865
Vertebrae	0.503	[0.301 0.854]	**0.009**
Etiological agents			
Aspergillus	0.651	[0.388 1.111]	0.11
Blastomyces	3.868	[0.780 70.124]	0.191
Candida	0.995	[0.554 1.875]	0.989
Coccidioides	0.767	[0.269 2.750]	0.646
Cryptococcus	2.042	[0.953 5.071]	0.089
Fusarium	0.268	[0.057 1.384]	0.089
Histoplasma	2.535	[0.488 46.537]	0.375
Mucormicosis	0.867	[0.402 2.089]	0.733
Paracoccidioides	-	-	-
Phaeohyphomycosis	-	-	-
Scedosporium	0.889	[0.375 2.459]	0.804
Other	0.501	[0.196 1.446]	0.168
**Outcome: Recurrence/chronicity (n=6)**			
**Variable**	**OR**	**95%CI**	**pvalue**
Surgical treatment	1.395	[0.756 2.725]	0.305
Treatment duration, months	1.087	[1.038 1.141]	**<0.001**
Time to diagnosis			
0 – 1 month	0.865	[0.491 1.501]	0.611
1 – 6 months	0.513	[0.275 0.917]	**0.028**
> 6 months	2.971	[1.576 5.468]	**<0.001**
Immunocompromised	0.66	[0.383 1.137]	0.134

Supplementary Table S6. Results of logistic regressions showing association of outcome (death, recovery, chronicity/recurrence) with variables of interest after elimination of articles of insufficient quality (54 in total, 3 for the death outcome, 32 for the recovery outcome, 1 for chronicity/recurrence outcome).

Site of infection			
Basicranium	0.371	[0.059 1.267]	0.181
Extremities (except foot)	0.952	[0.521 1.684]	0.87
Foot	0.922	[0.265 2.469]	0.885
Other cranium bones	1.022	[0.403 2.258]	0.96
Pelvis and hip	2.106	[0.897 4.546]	0.068
Ribs and sternum	1.626	[0.779 3.183]	0.172
Shoulder	2.752	[0.849 7.732]	0.066
Splanchnocranium	1.098	[0.481 2.226]	0.81
Vertebrae	1.586	[0.878 2.798]	0.117
Etiological agents			
Aspergillus	0.992	[0.531 1.784]	0.982
Blastomyces	0.727	[0.113 2.627]	0.676
Candida	1.160	[0.589 2.167]	0.651
Coccidioides	1.716	[0.476 4.927]	0.352
Cryptococcus	0.542	[0.202 1.221]	0.175
Fusarium	0.892	[0.047 5.142]	0.916
Histoplasma	4.174	[1.224 12.96]	**0.015**
Mucormicosis	0.832	[0.277 2.032]	0.713
Paracoccidioides	-	-	-
Phaeohyphomycosis	1.575	[0.079 10.870]	0.687
Scedosporium	1.173	[0.385 2.939]	0.752
Other	1.037	[0.237 3.186]	0.954
